# Temporal salt stress-induced transcriptome alterations and regulatory mechanisms revealed by PacBio long-reads RNA sequencing in *Gossypium hirsutum*

**DOI:** 10.1186/s12864-020-07260-z

**Published:** 2020-11-27

**Authors:** Delong Wang, Xuke Lu, Xiugui Chen, Shuai Wang, Junjuan Wang, Lixue Guo, Zujun Yin, Quanjia Chen, Wuwei Ye

**Affiliations:** 1 College of Agriculture, Xinjiang Agricultural University, 311 Nongda East Road, Urumqi, 830052 P. R. China; 2 State Key Laboratory of Cotton Biology/Key Laboratory for Cotton Genetic Improvement, Ministry of Agriculture/Institute of Cotton Research of Chinese Academy of Agricultural Science, Anyang, 455000 Henan China

**Keywords:** Differentially expressed genes, Upland cotton, Abiotic stress tolerance, Crop improvement, PacBio

## Abstract

**Background:**

Cotton (*Gossypium hirsutum*) is considered a fairly salt tolerant crop however, salinity can still cause significant economic losses by affecting the yield and deteriorating the fiber quality. We studied a salt-tolerant upland cotton cultivar under temporal salt stress to unfold the salt tolerance molecular mechanisms. Biochemical response to salt stress (400 mM) was measured at 0 h, 3 h, 12 h, 24 h and 48 h post stress intervals and single-molecule long-read sequencing technology from Pacific Biosciences (PacBio) combined with the unique molecular identifiers approach was used to identify differentially expressed genes (DEG).

**Results:**

Antioxidant enzymes including, catalase (CAT), peroxidase (POD), superoxide dismutase (SOD) were found significantly induced under temporal salt stress, suggesting that reactive oxygen species scavenging antioxidant machinery is an essential component of salt tolerance mechanism in cotton. We identified a wealth of novel transcripts based on the PacBio long reads sequencing approach. Prolonged salt stress duration induces high number of DEGs. Significant numbers of DEGs were found under key terms related to stress pathways such as “response to oxidative stress”, “response to salt stress”, “response to water deprivation”, “cation transport”, “metal ion transport”, “superoxide dismutase”, and “reductase”. Key DEGs related to hormone (abscisic acid, ethylene and jasmonic acid) biosynthesis, ion homeostasis (CBL-interacting serine/threonine-protein kinase genes, calcium-binding proteins, potassium transporter genes, potassium channel genes, sodium/hydrogen exchanger or antiporter genes), antioxidant activity (POD, SOD, CAT, glutathione reductase), transcription factors (myeloblastosis*,* WRKY*,* Apetala 2) and cell wall modification were found highly active in response to salt stress in cotton. Expression fold change of these DEGs showed both positive and negative responses, highlighting the complex nature of salt stress tolerance mechanisms in cotton.

**Conclusion:**

Collectively, this study provides a good insight into the regulatory mechanism under salt stress in cotton and lays the foundation for further improvement of salt stress tolerance.

**Supplementary Information:**

The online version contains supplementary material available at 10.1186/s12864-020-07260-z.

## Background

Salinity is one of the most limiting factors for plant productivity. Over 800 million hectares, equivalent to 6.5% of the world’s total land area, are currently estimated to be impacted by salinity [1]. Salt stress disturbs ion balance and osmotic homeostasis, leading to metabolic dysfunction and reduction in photosynthetic activity, finally resulting in reduction of crop productivity [2]. Plant salt stress response mechanism is mainly stimulated by osmotic stress and Na^+^ [3]. Plants employ various mechanisms to deal with salt stress; these mechanisms include minimization of the amount of salt taken up by roots and its partitioning at tissue and cellular levels to avoid buildup of toxic concentrations in the cytosol of functional leaves [4].

Plant’s physiological responses to salt stress involve a number of pathways, including hormone signaling transduction pathway, salt over sensitive pathway (SOS) and hormone biosynthesis pathways [5–9]. Phytohormones such as abscisic acid (ABA), ethylene (ET) and jasmonates (JA) play major roles against abiotic stresses. Abiotic stresses especially cold, heat and salinity are well known for inducing production of these hormones in plant under stress [6, 10, 11]. Salt stress also induces burst of oxidative stress by increasing production of reactive oxygen species (ROS). High ROS level causes molecular damage to DNA, proteins and lipids and also causes cell death in severe conditions [12–14]. Plants have developed antioxidant response mechanisms to scavenge this oxidative stress. Superoxide dismutase (SOD), peroxidase (POD) and catalase (CAT) are the main enzymatic components of this mechanism [15]. Molecular mechanisms of plant stress tolerance are much more complex than physiological and biochemical processes. Much efforts have been devoted to reveal the molecular mechanisms of plant salt tolerance, with the ultimate goal of improving salt tolerance of crop plants [16].

Although cotton (*Gossypium hirsutum*) is considered a relatively salt tolerant species, salinity can still have significant negative effects on its growth and productivity [17]. Cotton’s productivity and fiber quality are adversely affected by high salinity [18]. Salinity affects primary and secondary root development [19, 20], and limits photosynthesis and respiration, flowering, boll and fiber quality, and ion uptake in cotton, resulting in significant yield losses [21]. Expression levels of genes related to many biological processes and pathways are significantly affected by salt stress [22, 23]. The identification of salt tolerance genes is of key importance for improving cotton yield in salt affected lands [24, 25]. Limited number of salt tolerance genes have been identified in cotton as compared to other model plants [26, 27].

Genome-wide identification of salt stress related genes and regulatory pathways have been possible due to recent advances in high-throughput sequencing [7–9, 28–30]. Recently, a transcriptome study has revealed the molecular regulatory pathways to salt stress tolerance in cotton based on mRNA and miRNA networks in two contrasting cotton genotypes [16]. Unfortunately, we are still far behind in getting the complete understanding of salt stress tolerance and regulatory mechanisms, especially because of the limited time points investigated during salinity stress applications. Here, we investigated an upland cotton genotype under temporal salt stress. We compared all differentially expressed genes at four time-points with control to explore the plant’s response to salt stress under different time intervals. The results provided good insights into the regulatory pathways involved in response to salt stress in cotton.

## Results

### Biochemical response to salt stress in cotton

**Fig. 1:**
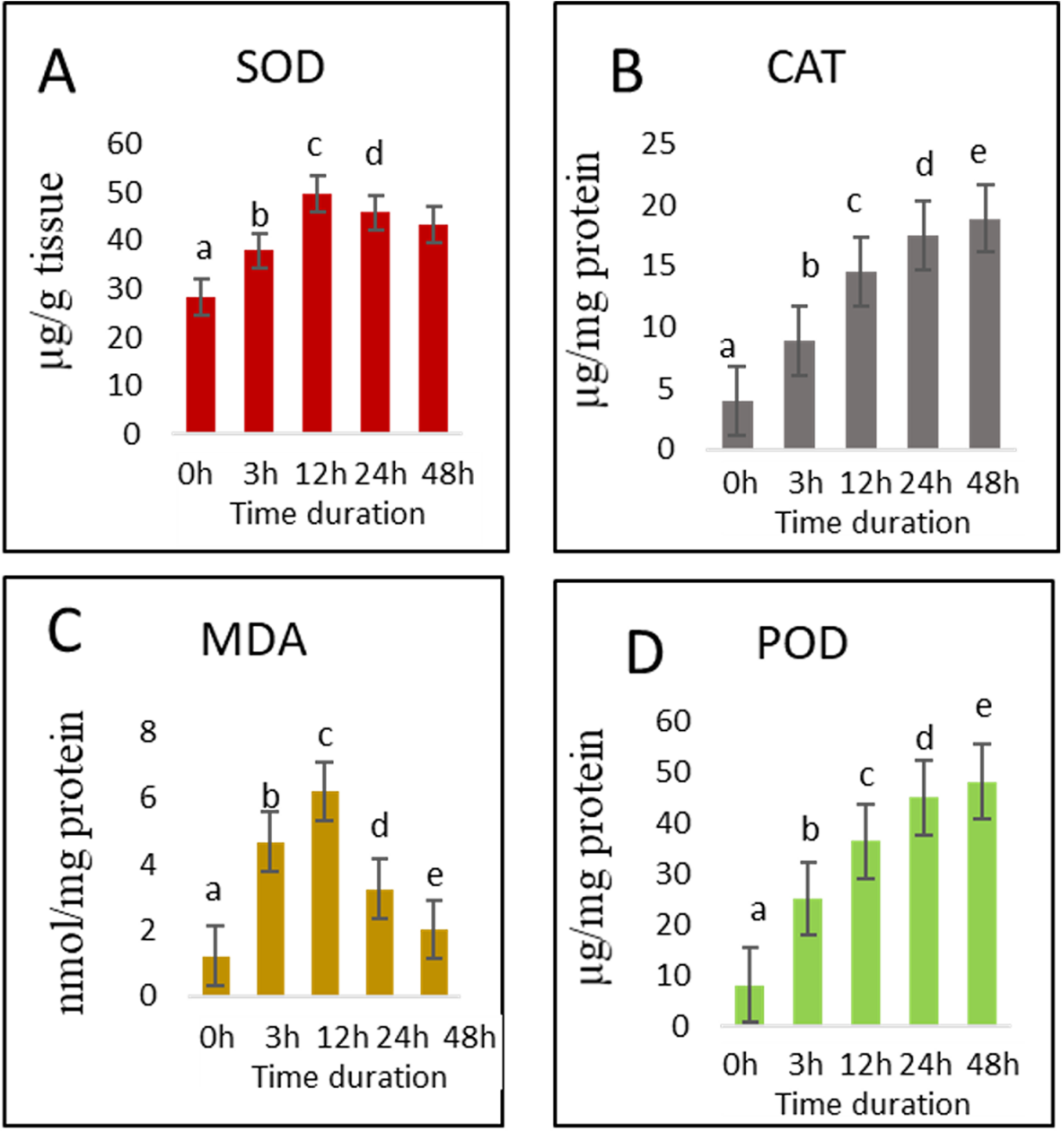
Biochemical and enzymatic activity under salt stress in cotton for (**A**) superoxide dismutase (SOD), (**B**) catalase (CAT), (**C**) malondialdehyde (MDA) and (**D**) peroxidase (POD). Data were measured at different time points under salt (400 mM) treatments. Letters above the bar (a, b, c) show the pair comparison. The bars sharing same letters represent groups which are statistically homogeneous. Error bars represent standard error

The cotton cultivar Zhong9807 which is a high salt-tolerant genotype [23, 31] was used in this study and subjected to different salt stress time durations (0 h, 3 h, 12 h, 24 h and 48 h). Under salt stress, excessive accumulation of reactive oxygen species (ROS) creates oxidative stress and in response, plants induce antioxidant response mechanisms by activating superoxide dismutase (SOD), peroxidase (POD), catalase (CAT) enzymes to scavenge ROS [15]. In this study, SOD, CAT, and POD were measured on young leaves collected at each time point under salt stress. CAT and POD showed continuous and significantly increased activity under temporal stress, while SOD activity showed a slight decrease after 12 h of stress (Fig. 1). However, SOD activity was significantly higher under salt stress conditions (3 h, 12 h, 24 h and 48 h) when compared to the control condition (0 h) (*P* < 0.05). Malondialdehyde (MDA) content is associated with lipid peroxidation via an increased generation of ROS [32]. Hence, a high level of MDA is an indicator of a high level of stress damage. Here, we found that the level of MDA increased until 12 h under salt stress, indicating that the plants experienced salt stress damage. However, after 12 h MDA level decreased sharply to reach a normal state as compared to the control condition (*P* = 0.19). This suggests that the sharp induction of antioxidant enzymes helped to scavenge ROS and led to cellular homeostasis. Therefore, we propose that ROS-scavenging antioxidant machinery is an essential component of salt tolerance mechanism in cotton.

### Processing and mapping of RNA-seq data

Fifteen sequencing libraries were constructed from five time points including, control (0 h) and four temporal salt stress treatments (3 h, 12 h, 24 h and 48 h) and used for single-molecule long-read sequencing on the Pacific Biosciences (PacBio) platform. .

A total of 174 Gb of data were generated with over a million of polymerase reads (Table S1). The adaptors and low-quality sequences were filtered out, resulting in a total of 98 million subreads. High quality reads of insert (ROIs) were further generated (8.6 million) after filtering CCS with full passes and accuracy. ROIs were classified as full length (FL) transcripts based on the presence of 5′ primers, 3′ primers and poly(A) tails. After polishing, clustering and demultiplexing of FL transcripts, on average we obtained 192,000 non-redundant high-quality FL non-chimeric (FLNC) transcripts with a mean length of 2400 bp in each library (Table S1). Isoforms were mapped to the reference genome and 98.55–98.87% of them were successfully mapped, whereas 1.25–1.45% isoforms were unmapped in each sample. Unique mapped isoforms ranged from 90.63–90.21%, while 68.64–74.96% of the isoforms were perfectly mapped (without any structural variation) to the reference genome (Table 1).

**Table 1:** Statistics of isoform mapping to the reference genome

Time points	Input	Mapped (%)	Unmapped (%)	Perfect mapped (%)	Unique mapped (%)	Multiple mapped (%)
0 h	380,361	98.6	1.4	68.64	90.63	7.97
3 h	400,895	98.85	1.15	70.94	91.2	7.65
12 h	377,903	98.67	1.33	71.53	91.17	7.49
24 h	350,330	98.55	1.45	68.06	91.21	7.34
48 h	383,056	98.75	1.25	74.96	91.19	7.56

### Overview of the alternative splicing events and effect of salt stress treatments

**Fig. 2:**
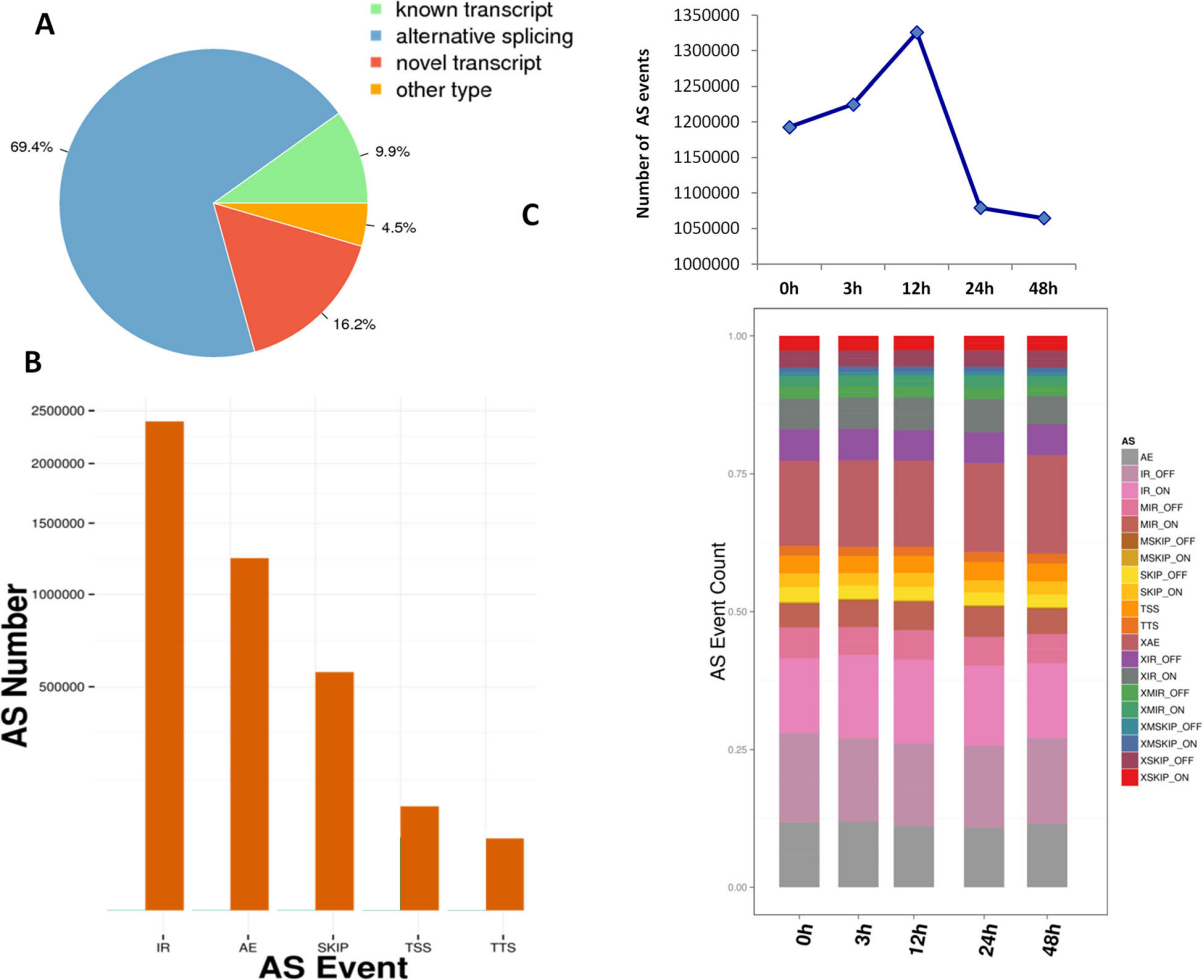
Classification of the isoforms and analysis of the alternative splicing events. **a** Classification of transcripts; **b** Distribution of the isoforms into five AS types; **c** Variation of the number of AS event over a temporal salt stress time treatment. Gene number according to the expression level (FPKM) at each time point. 0, 3, 12, 24 and 48 h represent the different time points of salt stress (400 mM) duration

A major advantage of PacBio sequencing is the possibility of identifying alternative splicing (AS) events by comparing isoforms of the same gene without de novo assembly. In this study, a total of 4,361,815 transcripts were classified into known transcripts, AS, novel and others. Known transcripts accounted only for 9.9% of the total transcripts while alternative spliced and novel transcripts accounted for 69.4 and 16.2%, respectively (Fig. 2a). This result highlights the importance of AS in cotton transcriptome particularly under salt stress treatment. The isoforms were classified into five AS types, including intron retention (IR), alternative exon (AE), exon skipping (SKIP), alternative transcript start and termination (TSS, TTS). As shown in Fig. 2b, IR and AE were the most abundant AS types in our experimental conditions. The number of AS types ranges from 2 to 436 with the gene *GH_D02G2617* having the highest number of isoforms (Table S2).

We further investigated the effect of salt stress treatments on AS profiles in cotton FL transcriptome. The number of AS events steadily increased from 0 h to 12 h under salt stress then, subsequently dropped until 48 h (Fig. 2c). This pattern is similar to the observed antioxidant enzyme activities under salt stress (Fig. 1). In more details, we observed that salt treatments not only affected AS number but also deeply influenced the AS types (Fig. 2c).

### Gene expression analysis at each time point

**Fig. 3:**
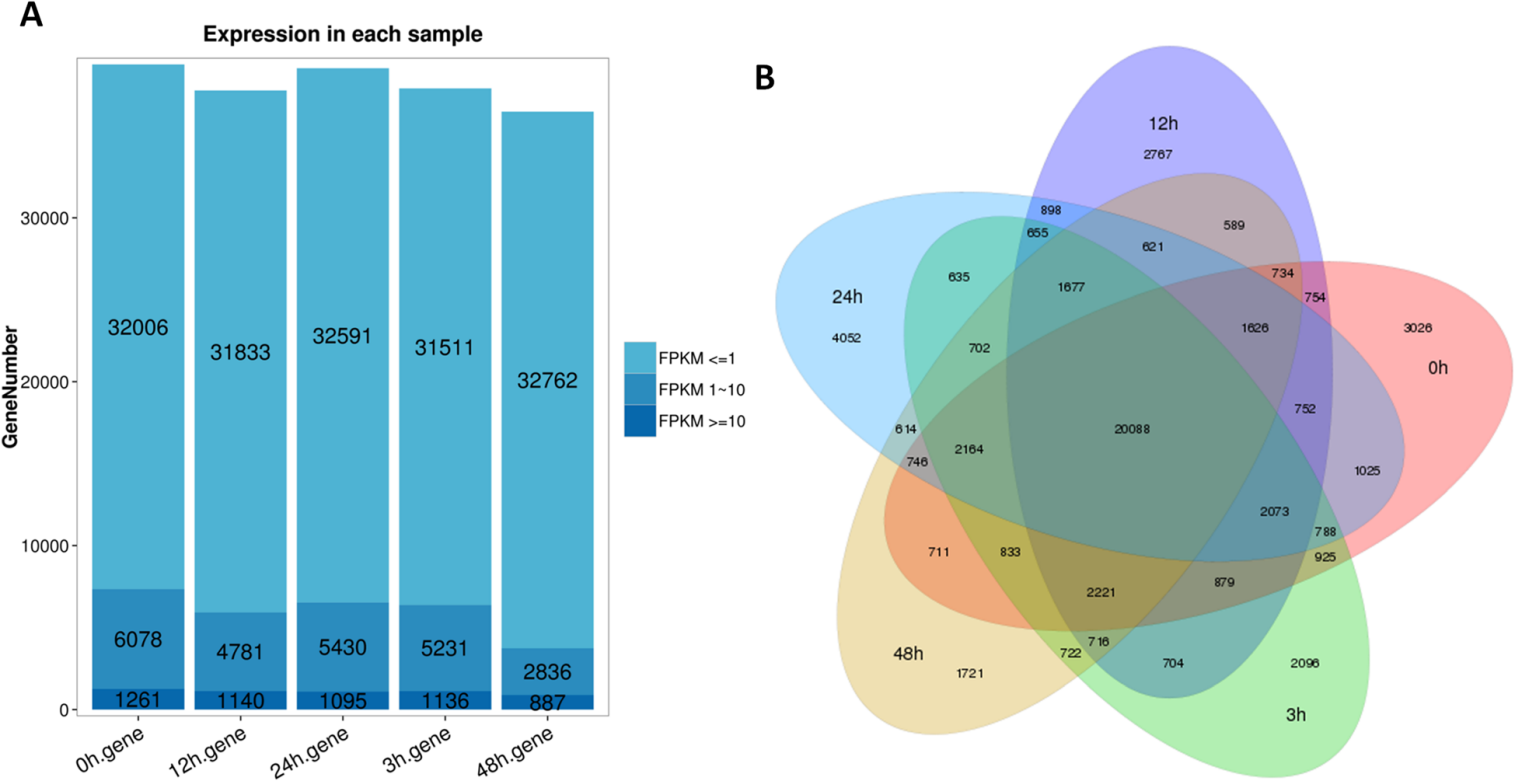
Gene expression profiles. **a** Gene number according to the expression level (FPKM) at each time point; **b** Venn diagram showing common and unique expressed genes at each time point. 0, 3, 12, 24 and 48 h represent the different time points of salt stress (400 mM) duration

Quantitative RNA-seq analysis was performed based on the unique molecular identifiers (UMI) approach described by Islam et al. [33]. Fragments Per Kilobase of transcript sequence per Millions of base pairs sequenced (FPKM) values were used to calculate the transcript abundance. The highest number of transcripts was expressed in control (0 h) sample while the lowest number of transcripts was expressed under 48 h salt stress sample (Fig. 3a), indicating that salt treatments globally reduce gene transcription in cotton. We constructed a venn diagram to show common and unique transcripts expressed among all samples. A total of 20,088 genes were commonly expressed among all samples and only few genes were time-specific (Fig. 3b).

Estimation of DEGs was done by using cut off *P* value of 0.05 adjusted by Benjamini and Hochberg’s approach and fold change ≥2. Results showed that the numbers of DEGs increased with prolonging salt stress duration (Table 2). We first tested the reliability of the DEG detection through a qRT-PCR gene expression profiling of 20 randomly selected DEGs. A high correlation between the qRT-PCR results and the transcriptome results (R^2^ = 0.79) showed that our DEG analysis were highly reliable (Table S3, Figure S1).

**Table 2:** Numbers of differentially expressed genes between compared time points

Time points	Total DEGs	up	down
0 h-vs-3 h	8444	3362	5082
3 h-vs-12 h	8673	3962	4711
12 h-vs-24 h	9541	5569	3972
24 h-vs-48 h	10,539	1880	8659

Then, we performed gene ontology (GO) and Kyoto Encyclopedia of Genes and Genomes (KEGG) pathway enrichment analyses to understand the functions of the DEGs. For GO terms, we annotated 5498 (65.11%), 5907 (65.85%), 5899 (60.56), 7529 (68.83%) DEGs for 0 h-vs-3 h, 0 h-vs-12 h, 0 h-vs-24 h and 0 h-vs-48 h, comparisons, respectively (Table 3). Down-regulated DEGs outnumbered the up-regulated DEGs in each GO category. We compared the DEGs under molecular function category between 0 h-vs-3 h and 0 h-vs-48 h. The numbers of genes showing differential expression in each GO term were higher under 0 h-vs-48 h than 0 h-vs-3 h. Three stress related GO terms (catalytic activity, transporter activity and antioxidant activity) were more enriched under 0 h-vs-48 h than 0 h-vs-3 h (Table S4). It shows that prolonged salt stress duration induces more genes for adjustment in the stressful environment compared to the non-stress condition.

**Table 3:** Gene ontology annotation of differentially expressed genes

Time points	up	down	total	% annotated
0 h-vs-3 h	2173	3325	5498	65.11
3 h-vs-12 h	2498	3266	5764	66.46
12 h-vs-24 h	3157	2560	5717	59.92
24 h-vs-48 h	1290	5007	6297	59.75

**Fig. 4:**
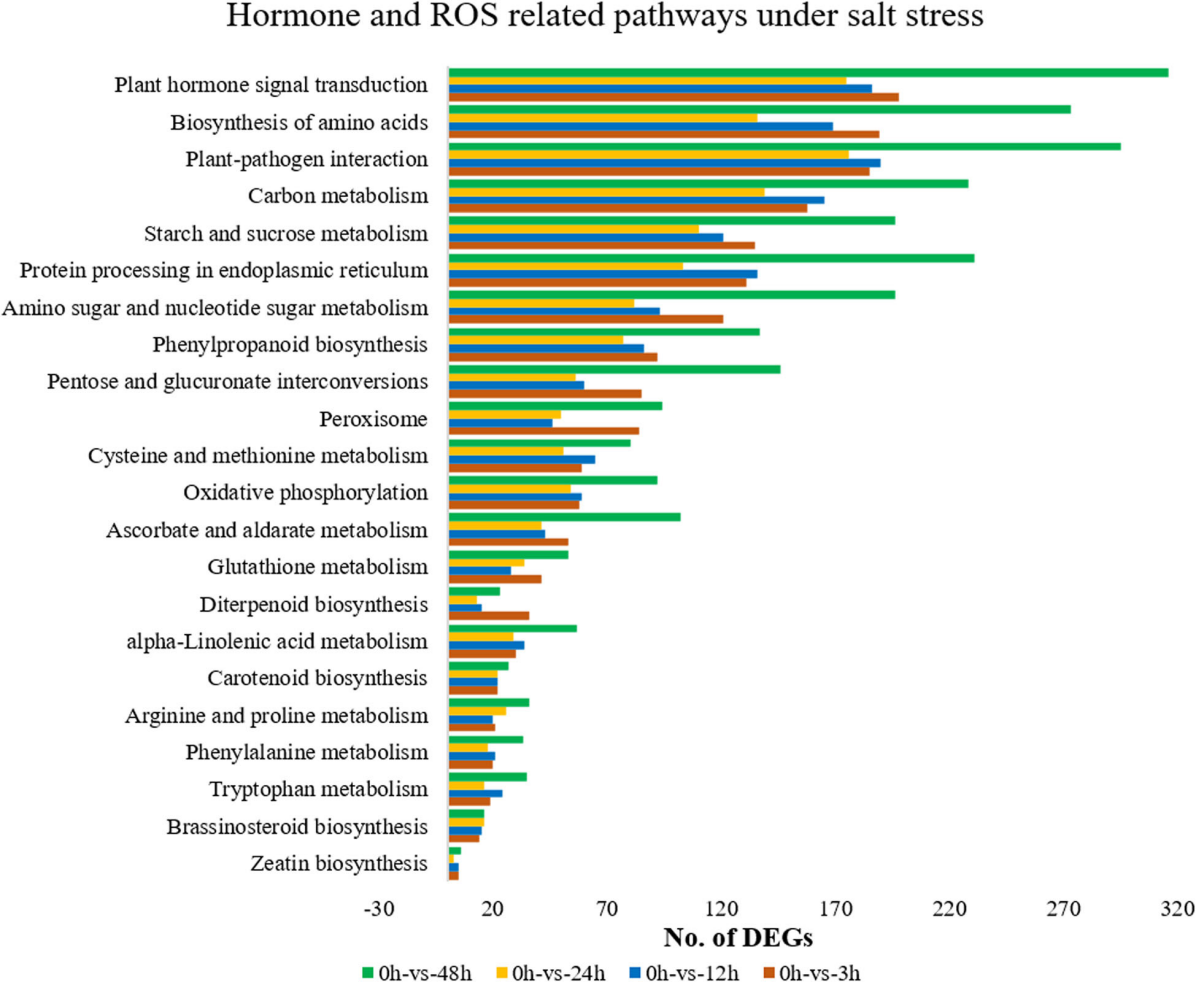
Hormones and ROS-related pathways under salt stress. 0, 3, 12, 24 and 48 h represent the different time points of salt stress (400 mM) duration

For further investigations of DEGs related to salt stress, we searched some key pathways related to stress. Significant numbers of DEGs were found under key terms such as “response to oxidative stress”, “response to salt stress”, “response to water deprivation”, “cation transport”, “metal ion transport”, “superoxide dismutase”, and “reductase” (Table 4). Based on the KEGG enrichment analysis, we observed that hormone synthesis, ROS related and hormone signal transduction related pathways were significantly enriched (Fig. 4; Table S5).

**Table 4:** Numbers of differentially expressed genes in each gene ontology (GO) term related to stress

GO term	0 h-vs-3 h	0 h-vs-12 h	0 h-vs-24 h	0 h-vs-48 h
Response to oxidative stress	18	16	10	16
Response to salt stress	8	11	9	7
Response to water deprivation	3	5	3	2
Cation transport	8	10	10	14
Metal ion transport	9	10	11	14
Superoxide dismutase	6	8	8	10
Reductase	34	26	31	33

### DEGs involved in hormone biosynthesis

**Fig. 5:**
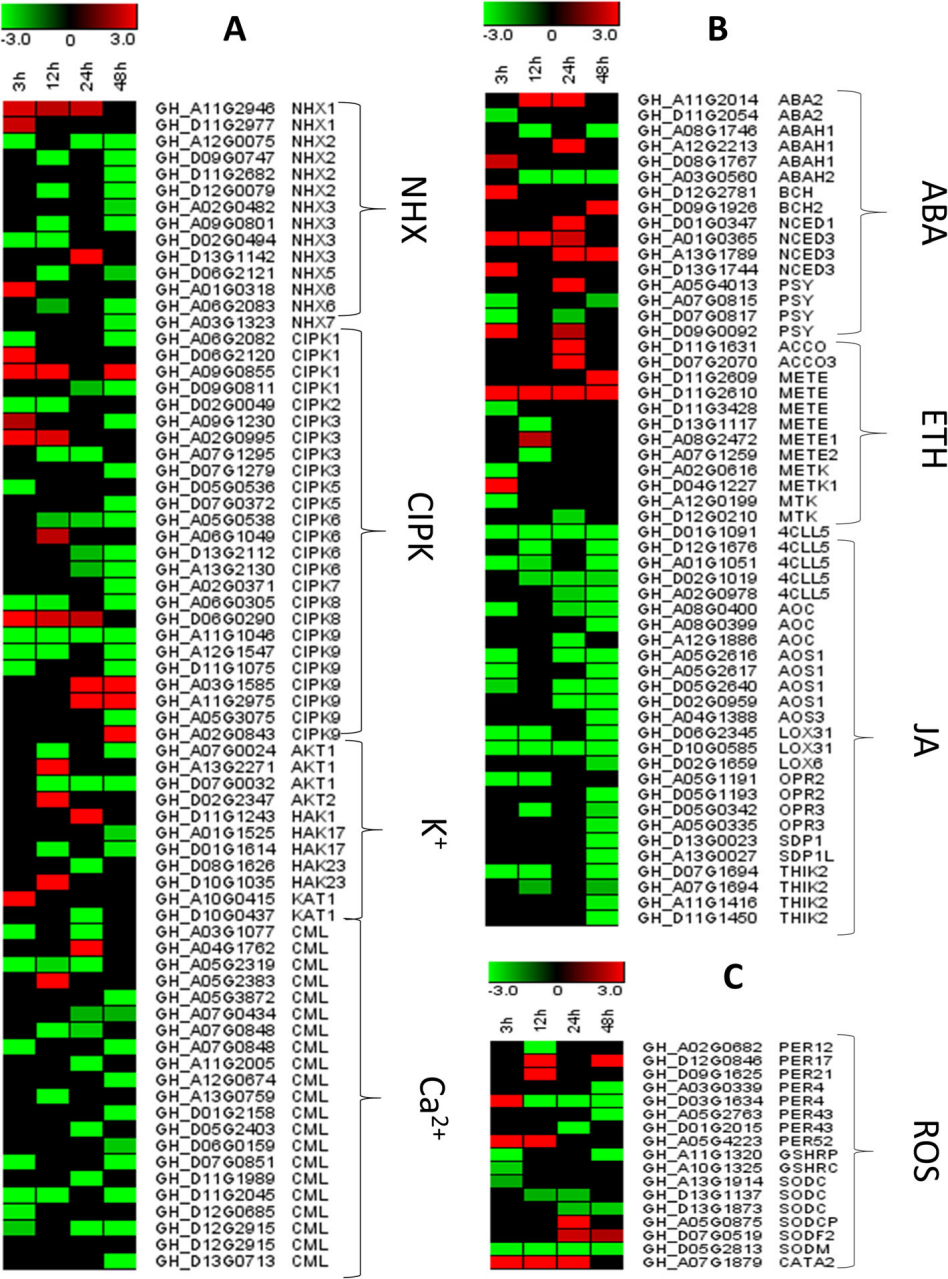
Expression profiling of ion homeostasis (**a**), hormone synthesis (**b**) and ROS related (**c**) DEGs. 0, 3, 12, 24 and 48 h represent the different time points of salt stress (400 mM) duration

Plant hormones play a crucial role in stress tolerance mechanism [6, 34]. Plant’s response to stress mainly depend on stress hormones for signal transduction [12]. Abscisic acid (ABA), ethylene (ET) and jasmonic acid (JA) are among the most important plant hormones known for long distance signal transduction during stress [11]. We examined KEGG pathways related to biosynthesis of these hormones. Two abscisic acid biosynthesis protein 2 (*ABA2*), three abscisic acid 8′-hydroxylase 1 (*ABAH1*), two beta-carotene hydroxylase (*BCH*), four 9-cis-epoxycarotenoid dioxygenase (*NCED*) and four phytoene synthase (*PSY*) genes showed differential expression in ABA biosynthesis pathway (Table S4, Fig. 5). *NCED* genes have been demonstrated to play an important role in the biosynthetic pathway of ABA [28]. Four *NCED* genes showed continuous up-regulation during all stress stages. In the JA biosynthesis pathway, 26 genes showed differential expression under salt stress. These genes include five 4-coumarate--CoA ligase-like 5 (*4CCL5*), three Allene oxide cyclase (*AOC*), four Allene oxide synthase 1 (*AOS1*), two Linoleate 13S-lipoxygenase 3–1 (*LOX*), four 12-oxophytodienoate Reductase 2 (*OPR*), two Triacylglycerol lipase (*SDP1)* and four 3-ketoacyl-CoA thiolase 2 (*THIK*) genes (Table S6, Fig. 5). Most of these genes were down-regulated under salt stress. In the ethylene biosynthesis pathway, 12 differentially expressed genes were found. Most of these genes showed positive regulation with the increase of stress duration. These results indicate that ABA, JA and Ethylene related DEGs play crucial roles for cotton response to salt stress.

### DEGs related to antioxidant activity

Salt stress leads to oxidation stress through over-accumulation of ROS. ROS is known for its negative effect on plant growth, productivity and other biological processes [13]. Plants have developed ROS scavenging mechanisms to avoid oxidative stress. Under stressful conditions, the balance between ROS production and scavenging is disturbed leading to over-accumulation of ROS, especially H_2_O_2_, in plant tissues [14]. Some antioxidant enzymes such as peroxidase (POD), superoxide dismutase (SOD), catalase (CAT) and glutathione reductase (GR) play an important role in ROS scavenging mechanism [10, 12]. We found eight genes from the POD family showing differential expression in our data. Four of these genes were up-regulated in salt stress conditions (Table S7, Fig. 5). One catalase gene (*GH_A07G1879*) was up-regulated under salt stress conditions. Six genes from the SOD family showed differential expression (Table S7, Fig. 5), with two up-regulated under salt stress conditions. Finally, we found two genes related to GR, both down-regulated under salt stress conditions. Differential expression of antioxidant related genes correlates well with the observed increased activity of antioxidant enzymes under salt stress except for SOD and POD. Some of the SOD and POD related genes were down-regulated with increasing salt stress duration, while POD and SOD enzymes showed continuous increased activities. This suggests that down-regulation of some POD and SOD genes did not affect the enzymatic activities under salt stress (Fig. 1).

### Ion homeostasis related DEGs

Ion balance is greatly damaged after exposure to salt stress [35–44]. High concentration of Na^+^ activates the Ca^2+^ signaling pathway. The role of Ca^2+^ as a secondary messenger is well known [15]. We found 25 CBL-interacting serine/threonine-protein kinase 1 (*CIPK*) genes showing significant differential expression under salt stress conditions (Table S8, Fig. 5). *CIPK* genes have been reported for playing a crucial role in salt overly sensitive (SOS) signaling and Ca^+^ signaling pathways [37]. We also found 21 calcium-binding protein (*CML*) DEGs (Table S6, Fig. 5). *CML* genes interact with *SOS* pathway and *CIPK* genes to activate the Ca^+^ signaling pathway [38]. Three DEGs encoding potassium transporter (*HAK*) were detected under salt stress (Table S8, Fig. 5). These genes are reported as potassium transporters [39–42]. Furthermore, we found six potassium channel (*AKT*, *KAT*) genes showing differential expression [43]. *AKT* and *KAT* are crucial potassium ion homeostasis. Sodium/hydrogen exchanger or antiporter genes (*NHX*) also play an important role in exclusion of excessive sodium ions under salt stress [44]. We found 14 *NHX* DEGs with various patterns of regulation under salt stress (Table S8, Fig. 5).

### Transcription factors related to salt stress

Transcription factors (TF) play key roles in gene regulation. Plants use transcription modulation in response to stress conditions [45, 46]. Stress responsive TFs modulate many genes related to ABA biosynthesis and signal transduction. Transcription families such as myeloblastosis (*MYB*), *WRKY* and *ERF* have been reported to regulate stress tolerance in plants [47]. In our data, 23 TF families containing 5723 members were detected. We found 26, 26, 18 and 35 TFs showing significant differential expression in 3 h, 12 h, 24 h and 48 h salt stress, respectively, compared to 0 h. Most of these TFs were members of *APETALA 2* (*AP2*), *WRKY* and *MYB* families (Table S9).

### DEGs related to cell wall modification

Salinity stress affects plant growth by inhibiting cell elongation and limiting cellulose synthesis. Cell wall becomes rigid and root growth gets affected [48]. Multiple regulatory processes come into action to resume the normal growth of the plant cells. Understanding the molecular mechanisms regulating cell wall integrity and modification could be useful for improving salt stress tolerance in plants. We found 235 DEGs related to cell wall biosynthesis and modification under salt stress (Table S8). Cellulose is one of main components of cell wall [49]. We found 50 DEGs related to cellulose biosynthesis. Most of these genes were down-regulated under slat stress. Only 19 genes were up-regulated (Table S10). We also found 17 DEGs related to cellulose catabolic process and 6 DEGs related to cellulose microfibril organization showing.

After cellulose, lignin is the second most abundant compound in plants. It gives plants the ability to stand. Lignification of plant cell wall is affected under various abiotic stresses [50]. We found seven DEGs related to lignin catabolism under salt stress (Table S10). All of these genes were up-regulated. Expansins play important role in cell elongation by loosening the cell wall [51]. We found 21 expansin genes showing differential expression under salt stress. Only five out of the 21 expansin DEGs were up-regulated. We also found one extensin gene (*EXTN*) down-regulated under salt stress (Table S10). Pectin is another important component of cell wall structure. Under various abiotic stresses, pectin can stiffen the cell wall and slow down the growth [48]. We found 42 DEGs related to pectin catabolic process under salt stress. Only 12 out of the 42 DEGs were up-regulated (Table S10). Galacturonosyltransferases are important enzymes involved in pectin biosynthesis [48]. We found 28 DEGs related to galacturonosyltransferase activity under salt stress (Table S10). Osmotic stress can induce peroxidases in plants. Peroxidases play role in cross-linking various cell wall structural proteins [52]. We found seven DEGs related to peroxidase activity under salt stress. Two of these genes were significantly up-regulated. Receptor-like kinases have also been proved to play important role in cell wall modification under salt stress [53]. We found seven receptor-like kinase genes showing significant differential expression under salt stress (Table S10). Xyloglucans are hemi cellulosic polymers present in cell wall structure. They play role in cell wall organization [48]. In our experiment, we found 37 genes related to xyloglucan showing differential expression. Thirteen out of the 37 DEGs were up-regulated. Nine out of ten genes related to cell wall structural proteins were up-regulated under salt stress. One gene encoding for leucine-rich repeat extensin like protein was down-regulated (Table S10).

## Discussion

Salinity stress negatively affects plant growth and other developmental processes. Salinity induces osmotic stress and ionic imbalance leading to ion toxicity and production of reactive oxygen species (ROS) [10]. After receiving the stress stimulus, receptors activate secondary signaling pathways such as Ca^+^, ROS and hormone signaling [13]. These signal molecules activate salt stress tolerance mechanism related genes. Although cotton is moderately tolerant to salt stress, its salt tolerance mechanism is not well understood [54]. In this study, we applied temporal salt stress to cotton plants and used PacBio long reads combined with the unique molecular identifiers approach [33] to examine differential expression of genes at each time point. We identified a wealth of novel transcripts based on the PacBio long reads sequencing approach, which is accordance with previous report of Wang et al. [55]. We compared four time points of varying stress duration with control. A significant number of DEGs were found under salt stress at each time point. We focused on hormone biosynthesis, ion homeostasis and antioxidant related pathways to further investigate salt tolerance mechanism in cotton.

Phytohormones, especially ABA, play major role of signal transduction under abiotic stress [56]. Increased level of ABA in plant under salt stress promotes membrane stability and Ca^+^ uptake [34]. In this study, ABA biosynthesis genes (*ABA*, *BCH*, *NCED*, *PSY*) were activated during early stages of stress and showed continuous up-regulation at all time points. Genes related to ABA degradation also showed differential expression. Some of these genes were up-regulated at late stages of stress, which implies that activation of these genes was in response to balance the ABA content in the plant tissues. Regulation of ABA biosynthesis genes under salt stress depicts their crucial role in stress tolerance mechanism. A considerable number of ethylene and JA related genes were also activated at early stress stage and showed continuous differential expression at all time points. Various studies have reported the role of endogenous ethylene production or treatment with ethylene in enhancing salinity tolerance [57, 58]. Some studies have also reported negative effect of higher level of ethylene on salt tolerance in plants [59, 60]. These results suggest that ethylene regulates salinity tolerance negatively or positively according to the endogenous concentration and optimising the ethylene production in plants can enhance salt stress tolerance.

Salt stress increases Na^+^ concentration in the cytosol. Plant uses Na^+^/H^+^ exchanger (NHX protein) to regulate Na^+^ concentration [61–63]. NHX proteins located in plasma membrane pump out excessive Na^+^ ions from cell, while NHX proteins located in the tonoplast direct the Na^+^ ion into the vacuole and maintain ion homeostasis in the cytosol [14]. The SOS signaling pathway has been well documented for playing preponderant role in ion homeostasis [64]. Salt stress changes the free Ca^+^ concentration in the cytosol. Change in Ca^+^ concentration activates SOS3 and SOS2. SOS3-SOS2 complex brings Na^+^/H^+^ exchanger into action and the excess Na^+^ is excluded from the cytosol [38]. Potassium channels and transporters also play key role in ion homeostasis [65]. In the present study, three sodium/proton antiporters were up-regulated. Potassium transporters and channels (*AKT, HAK, KAT*) and SOS pathway related genes (*CIPK, CML*) showed significant differential expression under salt stress. Regulation of these genes under salt stress is consistent with previous studies [29, 65–69]. Although many studies have proved the role of ion homeostasis related genes in salt stress tolerance, the interaction of these genes to confer the stress tolerance is yet to be explored.

Salt stress also induces bursts of oxidative stress by increasing the production of ROS [70]. High ROS level causes molecular damage to DNA, proteins and lipids, resulting in cell death under severe conditions [12–14]. Plants have developed antioxidant response mechanism to scavenge this oxidative stress. Superoxide dismutase (SOD), peroxidase (POD) and catalase (CAT) are the main enzymatic components of this mechanism [15]. In our study, biochemical analysis of the antioxidant enzymatic activity showed a steep increase of the levels of most of the enzymes under salt stress in cotton, indicating that the cotton genotype triggered the antioxidant machinery (CAT, POD, SOD) to buffer ROS production. Although the MDA content increased during the early salt stress stage, it rapidly decreased to reach the normal range after 12 h. This indicates that the enzymatic ROS scavenging machinery was highly efficient to buffer the salt stress damage in cotton. Similarly, eight genes from the POD family, one CAT gene (*GH_A07G1879*) and six genes from the SOD family showed differential expression under salt stress based on the RNA-seq data. These results are consistent with previous study in which overexpression of *GhSOD1* and *GhCAT1* increases salt stress tolerance in cotton plants [71]. Regulation of genes related to antioxidants has already been proved in previous studies to play an important role in salt stress tolerance [72–74]. Together with other signaling pathways, ROS scavenging mechanism confers salt stress tolerance [75].

Transcription factors (TF) are very important elements for salt stress tolerance. Various transcription families have been characterised and their role in stress tolerance mechanism has been proved. *WRKY* transcription factors have been reported to play an important role in salt stress tolerance mechanism [76, 77]. *MYB* transcription factors have also been reported in rice to modulate gene expression under salinity stress [36]. Our study reports 24 TF families showing differential expression under salt stress. Among these *WRKY, MYB, MYB-related, AP2-EREFF* and *GRAS* (*GAI, RGA, SCR*) TF families had highest number of DEGs. Three members of *bHLH* family were up-regulated under salt stress. Various members of *bHLH* family (*VvbHLH1*, *CgbHLH001*, *EcbHLH57*, and *TbHLH39*) enhance salt stress tolerance in transgenic plants [78–81]. Future studies on the identification of the major regulators within these TFs using approaches such as gene co-expression analysis will provide crucial tools to further enhance salt tolerance and improve productivity in cotton.

Cell wall in plant provides hardness and strength to stand against gravity and its chemical composition change during the course of growth and development. Cell wall is mainly composed of celluloses, hemicelluloses, pectin, lignin and some structural proteins [48]. Abiotic stresses have a significant effect on the cell wall structure. Cell elongation can be limited under abiotic stress due to stiffening of the cell wall or limited availability of celluloses [52]. Xyloglucan endotransglucosylase/hydrolase (*XTH*) and expansins play important role in cell wall organisation [51]. Understanding the regulatory mechanisms responsible for cell wall remodelling under salt stress can be valuable for the improvement of salt stress tolerance mechanisms. Our study revealed some key genes related to cell wall remodelling, differentially expressed under salt stress. Most of the genes related to cellulose biosynthesis and catabolic process were down-regulated under salt stress. A significant number of expansins and xyloglucan genes were up-regulated under salt stress. These genes are supposed to play important role in resuming the normal growth by loosening the cell wall and promoting elongation.

**Fig. 6:**
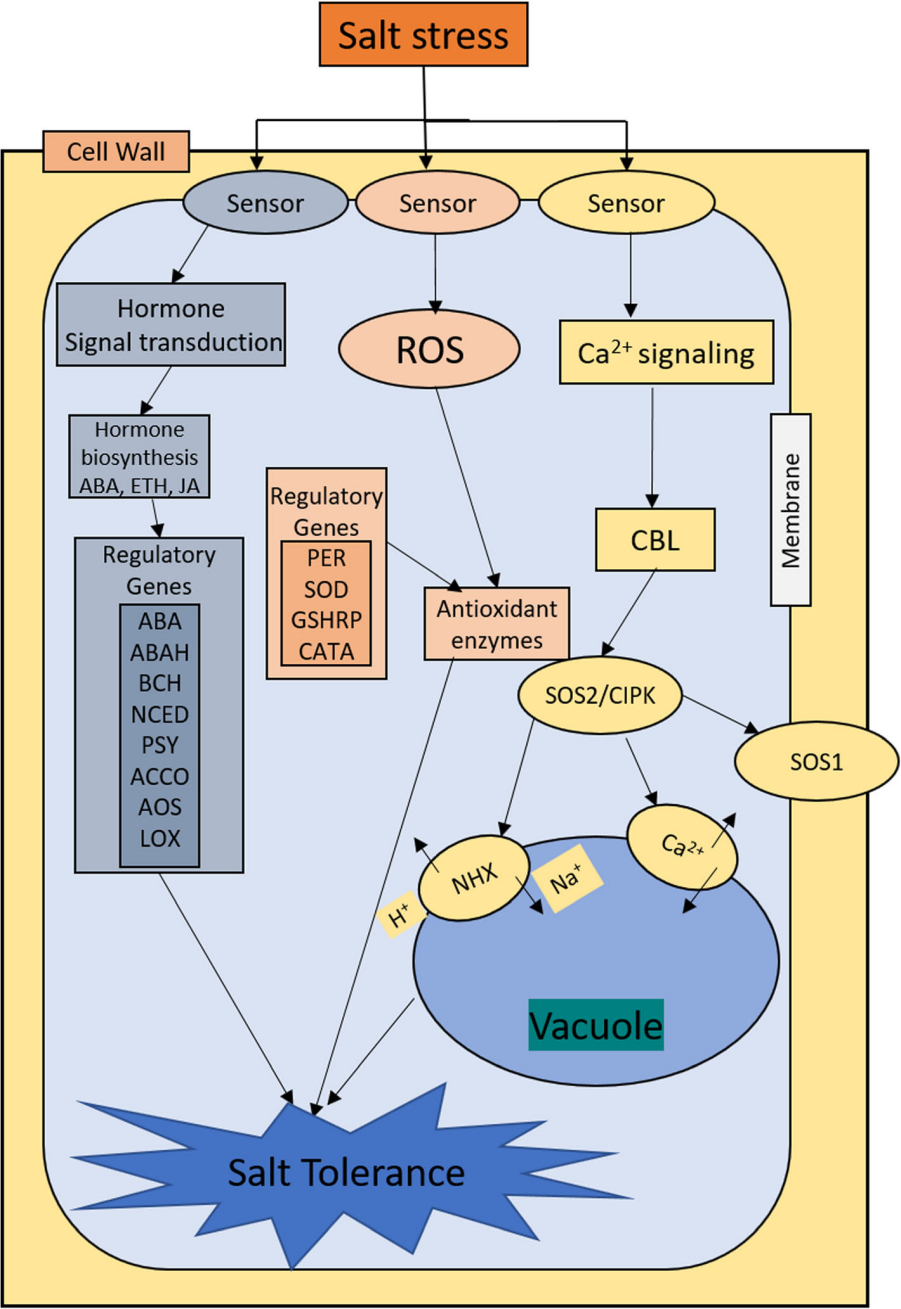
Hypothetical salt stress tolerance mechanism in cotton

Based on our findings and previous work of Deinlein et al. [82], we have proposed a hypothetical salt stress tolerance network in cotton (Fig. 6). Salt stress stimulus is received by sensors in plasma membrane. Hormone synthesis is regulated and hormonal signal transduction activates stress tolerance mechanism by activating relevant regulatory genes. ROS production is stimulated with the induction of salt stress. Antioxidant enzymes came into action to start ROS scavenging process. Salt stress also activates Ca^2+^ signaling. *CBL/CIPK* interaction activates *SOS1* which leads to exclusion of excessive Na^+^ ions. Sodium/proton antiporter also came into action and *NHX* proteins present in the tonoplast start pumping excessive Na^+^ ion from the cytosol to the vacuole. Although this hypothetical mechanism gives a good understanding of salt stress tolerance mechanism, more in-depth studies are needed to unfold the complex regulatory mechanisms and propose validated genes for salt tolerance improvement in cotton.

## Conclusions

We studied the molecular response of an upland cotton genotype under temporal salt stress based on long reads transcriptome analysis. Our study reports a wealth of novel transcripts and some key genes showing differential expression under salt stress. Hormone biosynthesis pathways were observed to play active role under salt stress. We also found sodium/proton antiporter genes, potassium and calcium channels, and SOS pathway related genes showing differential expression under salt stress. DEGs related to hormone biosynthesis (*ABA*, *NCED*, *PSY*, *AOC*, *SPD1*), ion homeostasis (*CIPK*, *CML*, *HAK*, *AKT*, *NHX*), antioxidant activity (*POD*, *SOD*, *CAT*, *GR*), transcription factors (*MYB*, *WRKY*, *AP2*) and cell wall modification were implicated in response to salt stress. Overall, this study provides a good insight into the complex molecular mechanisms of salt stress response in cotton and lays the foundation for further improvement of salt stress tolerance.

## Methods

Seeds of *Gossypium hirsutum* cv. Zhong 9807, a high salt-tolerant genotype [23, 31], were obtained from the gene bank of the Institute of Cotton Research, Chinese Academy of Agricultural Sciences, China and were grown in a plant growth chamber. The formal identification of the plant material was undertaken by the corresponding author of this article (Professor Wuwei Ye). The plant material has been deposited at the gene bank of the Institute of Cotton Research, Chinese Academy of Agricultural Sciences, under the accession ID: xcy2399. For the plant growth conditions, the day/night temperature was set at 28 °C/25 °C, the relative humidity was kept at 60–80%, and the light intensity was set at 14 h/10 h light/dark cycle under 450 μmol m^− 2^ s^− 1^. The seeds were sterilized with 3% sodium hypochlorite for 10 min and washed three times using sterile water. The seeds were germinated in a bowl containing sterilized sand soaked with 100 mL of ¼ Hoagland nutrient solution. Each bowl contains a single seedling and bowls were watered regularly to keep optimum soil moisture. A preliminary experiment was performed with different salt concentrations (0 mM, 200 mM, 250 mM, 300 mM, 350 mM, 400 mM) and a treatment time of 3 h to choose the optimum salt concentration for inducing salt stress in Zhong 9807 (Figure S2). Salt concentration of 400 mM was finally selected to induce salt stress. It is also important to mention that the seedlings could not survive under concentrations higher than 400 mM. When plants grew to 2–3 true leaves stage, 200 mL (400 mM) of salt (NaCl) solution was applied to the bowl. Treatment times were prior salt stress treatment (0 h), and 3 h, 12 h, 24 h and 48 h post salt stress treatment. Young leaves were harvested in triplicate (three different plants) at every time point, immediately placed in liquid nitrogen and stored at − 80 °C until use for RNA extraction and to measure the enzymatic activities.

### Measurement of enzymatic and biochemical parameters

Catalase (CAT), Malondialdehyde (MDA), Peroxidase (POD) and Superoxide dismutase (SOD) were measured using Nanjing Jiancheng Bioengineering Institute’s relevant kits following manufacturer’s instructions and well detailed by Tang et al. [83]. Peroxidase (POD, E.C. 1.11.1.7) activity was assayed by peroxidase assay kit (Catalog No.A084; Jiancheng Bioengineering Institute, Nanjing, China). POD can catalyze the reaction of hydrogen peroxide, the enzyme activity of POD was obtained by measuring the change of absorbance at 420 nm. Superoxide dismutase (SOD, E.C. 4 1.15.1.1) activity was measured using a superoxide dismutase activity assay kit (Catalog No. A001–1; Jiancheng Bioengineering Institute, Nanjing, China). SOD activity was determined by the xanthine oxidase method (hydroxylamine). Catalase (CAT, E.C. 1.15.1.1) activity was measured using a CAT activity assay kit (Catalog No. A007–1; Jiancheng Bioengineering Institute, Nanjing, China). CAT can decompose H_2_O_2_ and this reaction can be quickly suspended by adding ammonium molybdate, the rest of H_2_O_2_ combine with ammonium molybdate to produce a pale-yellow complex compound, which is detected at 405 nm. Malondialdehyde (MDA) was measured using the *MDA* assay *kit* (TBA method) (Catalog No. A003–1; Jiancheng Bioengineering Institute, Nanjing, China) The MDA content was measured at 532 nm in nmol mg^− 1^ proteins.

### Statistical analysis

Data were analyzed with the R software (www.r-project.org) using the one-way analysis of variance (ANOVA) for significant difference. The error bars were calculated with data from three replicates. ANOVA results were considered significant at *P* < 0.05 and mean comparisons were done using the Tukey HSD test.

### Library preparation and PacBio sequencing

Plant leaf tissues at 3-leaves stage were collected for total RNA extraction to construct UMI (unique molecular identifiers) IsoSeq PacBio sequencing libraries following PacBio instructions. Clontech SMARTer PCR cDNA Synthesis Kit and UMI primers were used to synthesize UMI + cDNA. 1 + 0.4X AMPure PB beads were used for fragment screening to construct standard full-length transcriptome library. PCR amplification by KAPA HiFi PCR Kits was followed by size selection of PCR product by agarose gel electrophoresis. These cDNA products were purified for library construction using SMRTbell template prep kit 1.0. Libraries were sequenced using P6C4 polymerase and chemistryon PacBio RS II platform with 240 min movie time. In this project, we built three UMI IsoSeq PacBio libraries per time point (0 h, 3 h, 12 h, 24 h and 48 h). (More about the library construction protocol detail and performance can be found in this wiki: https://github.com/shizhuoxing/BGI-Full-Length-RNA-Analysis-Pipeline/wiki).

### Quality control

Data processing after Pacific Biosciences Sequel was performed with SMRT analysis software suite (http://www.pacb.com/products-and-services/analytical-software/smrt-ana lysis/) for reads of insert (ROI), Reads classification, Reads clustering and correction (Cluster, Quvier), resulting in high-quality full-length consistent sequences. Briefly, raw polymerase reads were filtered and trimmed to generate the subreads and read of inserts (ROIs) using the RS Subreads protocol, requiring a minimum polymerase read length of 50 bp, a minimum polymerase read quality of 0.75, a minimum subread length of 50 bp, and a minimum of one full pass. (Dependencies: SMRTlink v8.0/ ncbi-blast v2.2.26+/ R v3.4.1 with ggplot2 v3.3.0 and gridExtra v2.3.)

### Mapping and sequence annotation

*Gossypium hirsutum* reference genome [84] and its annotation files were used as background data for the analysis of our transcriptome data. We used GMAP (v2015-09-29) with the parameters “-K 20000 -B 4 -f 2”) [85] to align the filtered reads with the reference genome and the result output file was stored in SAM format. Coding sequences (CDS) were identified using Transdecoder [86]. We used the Cuffcompare utility of the Tuxedo suite [87] to categorize each long-read transcript with respect to its most closely matching reference transcript. We used Blastn (http://blast.ncbi.nlm.nih.gov/Blast.cgi/v2.2.23) to annotate the Isoforms with NT; Blastx and Diamond (https://github.com/bbuchfink/diamond/v0.8.31) were employed to annotate the Isoforms with NR, KOG, KEGG and SwissProt; Blast2GO (https://www.blast2go.com/v2.5.0) [88] and NR annotation results were used to annotate the Isoforms with GO. The related parameters could be found at this link (https://github.com/shizhuoxing/BGI-Full-Length-RNA-Analysis-Pipeline).

### Gene expression quantification and differential expression analysis

The UMI sequence tagging passed reads was captured according to the positions of the polyA tail and the 3′ primer sequence using blast:-outfmt 7-word_size 5. More details could be found at this link (https://github.com/shizhuoxing/BGI-Full-Length-RNA-Analysis-Pipeline/). Fragments Per Kilobase of transcript sequence per Millions of base pairs sequenced (FPKM) value of each gene was computed based on the UMI-count method described by Islam et al. [33]. In this study, genes with FPKM > 1 were considered expressed.

We used PossionDis (R v3.4.1/ Fold Change > = 2.00 and False Discovery Rate (FDR) < = 0.001 [89]) to screen differentially expressed genes (DEGs) between two samples by referring to the sequencing based differential gene detection method published by Audic et al. [90].

### GO and KEGG enrichment analysis of DEGs

DEGs were also employed for the enrichment analysis of GO using the Blast2GO V2.5.0 [88], which can adjust the gene length bias. The adjusted *P*-value of significantly substantiated GO terms was less than 0.05. KOBAS V2.0 software was used to detect the KEGG pathways enriched with DEGs [91]. The standard of significantly enriched pathway is the same as GO enrichment.

### Validation of RNA-seq by qRT-PCR

Real-time RT-PCR was performed on three replicates of each sample following descriptions of Dossa et al. [92]. Twenty genes were randomly selected for qRT-PCR. NCBI blast was used to design specific primers for the selected genes. Real-time PCR was performed on EDC-810 system (Dongsheng innovation Biotechnology Co., Ltd) using SYBR Green Master Mix and results were analyzed by ΔΔCt method. *Actin* gene (F: ATCCTCCGTCTTGACCTTG, R: TGTCCGTCAGGCAACTCAT) was used as control. Each reaction was carried out in a final volume of 20 μL containing 10 μL of SYBR Green master mix, 0.4 μL of each of primer for selected gene and 4 μL of cDNA. The PCR thermal cycling conditions were as follows: 95 °C for 10 min; 40 cycles of 95 °C for 5 s, 60 °C for 30 s and 72 °C for 30 s. Data were collected during the extension step: 95 °C for 15 s, 60 °C for 1 min, 95 °C for 30 s and 60 °C for 15 s.

## Supplementary Information

**Additional file 1: Figure S1.** qRT-PCR result and correlation analysis with RNA-seq of some selected genes. The cotton *Actin* gene was used as endogenous gene for normalisation. The error bar represents the SD of three biological replicates.

**Additional file 2: Figure S2.** Effect of various concentrations of salt (NaCl) on cotton plant (stress time duration was 3 h).

**Additional file 3: Table S1.** Statistics of the PacBio sequencing and data processing.

**Additional file 4: Table S2.** Statistics of the isoforms, corresponding genes and alternative splicing events.

**Additional file 5: Table S3.** Primer list of transcripts for real-time qRT-PCR.

**Additional file 6: Table S4.** GO enrichment analysis of the differentially expressed genes.

**Additional file 7: Table S5.** KEGG pathway enrichment of differentially expressed genes.

**Additional file 8: Table S6.** Differentially expressed genes related to hormone biosynthesis.

**Additional file 9: Table S7.** Differentially expressed genes related to ROS production and scavenging.

**Additional file 10: Table S8.** Differentially expressed genes related to ion homeostasis.

**Additional file 11: Table S9.** Differentially expressed transcription factors under salt stress.

**Additional file 12: Table S10.** Differentially expressed genes related to cell wall modification under salt stress.

## Data Availability

The raw RNA-seq datasets supporting the conclusions of this article are available in the National Center for Biotechnology Information Bioproject repository, accession number: PRJNA559592 (https://www.ncbi.nlm.nih.gov/bioproject/?term=PRJNA559592). The reference genome of *Gossypium hirsutum* is available at http://ibi.zju.edu.cn/cotton/.

## Abbreviations

PacBio: Pacific Biosciences
DEG: Differentially Expressed Genes
ABA: Abscisic Acid
SOS: Salt Over Sensitive
ROS: Reactive Oxygen Species
SOD: Superoxide Dismutase
POD: Peroxidase
CAT: Catalase
MDA: Malondialdehyde
FPKM: Fragments Per Kilobase of transcript sequence per Millions of base pairs sequenced
GO: Gene Ontology
KEGG: Kyoto Encyclopedia of Genes and Genomes
GR: Glutathione Reductase
TF: Transcription Factor
ANOVA: Analysis Of Variance
