# Supplementary material for: Temporal salt stress-induced transcriptome alterations and regulatory mechanisms revealed by PacBio long-reads RNA sequencing in Gossypium hirsutum
Source: BMC Genomics. 2020 Nov 27;21:838. doi: 10.1186/s12864-020-07260-z (PMC7694341; doi:10.1186/s12864-020-07260-z)
Supplement: Supplementary file 1 — Additional file 1: Figure S1. qRT-PCR result and correlation analysis with RNA-seq of some selected genes. The cotton Actin gene was used as endogenous gene for normalisation. The error bar represents the SD of three biological replicates. [file 12864_2020_7260_MOESM1_ESM.docx]

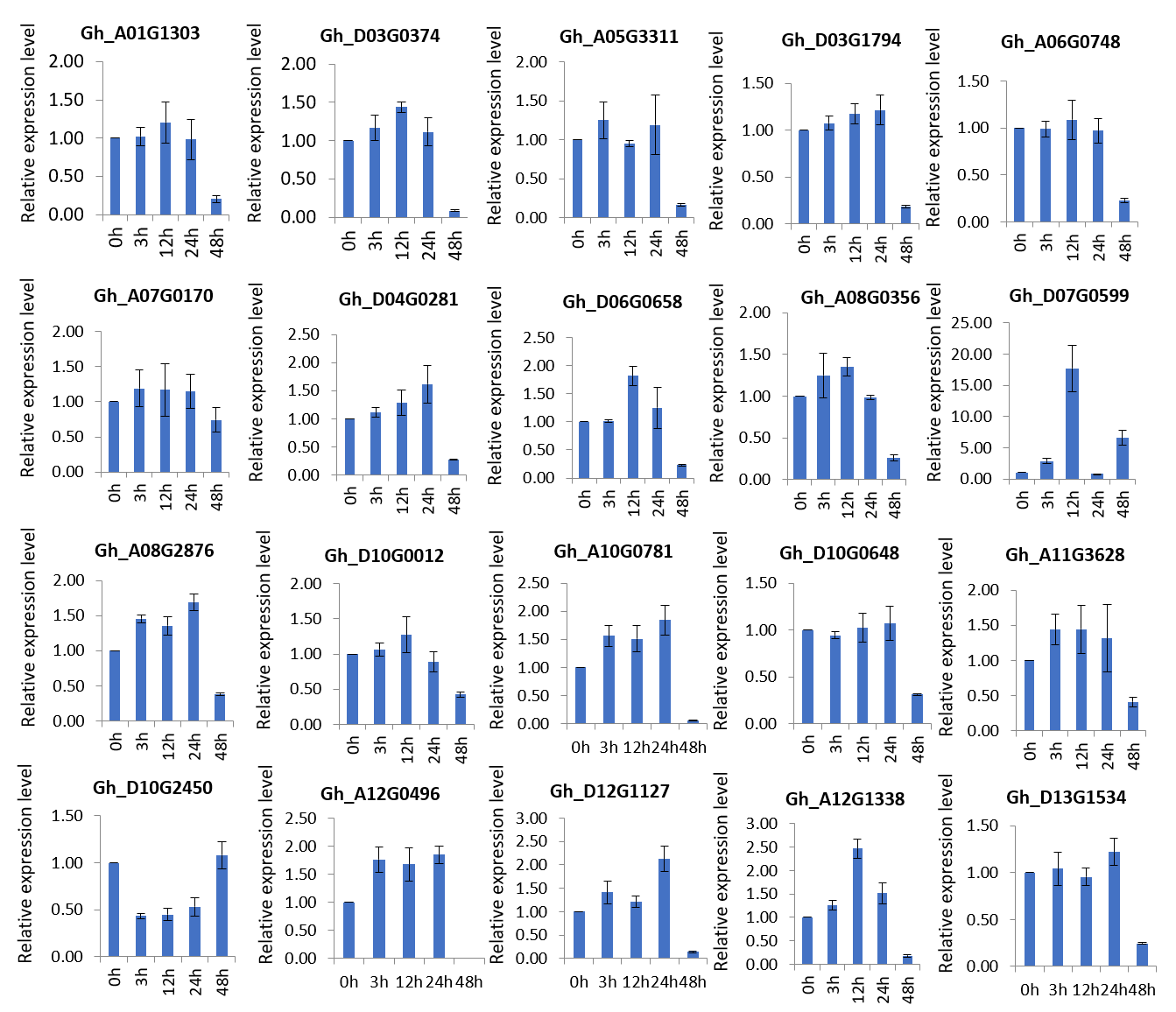


**Figure S1** qRT-PCR validation of selected genes and correlation with transcriptome. The *Actin* gene was used as reference for normalization. 0 h, 3 h, 12 h, 24 h, 48 h represent samples collected at different time points under salt stress (400 mM). The error bars represent standard deviation of three biological repeats.
