# Supplementary material for: Temporal salt stress-induced transcriptome alterations and regulatory mechanisms revealed by PacBio long-reads RNA sequencing in Gossypium hirsutum
Source: BMC Genomics. 2020 Nov 27;21:838. doi: 10.1186/s12864-020-07260-z (PMC7694341; doi:10.1186/s12864-020-07260-z)
Supplement: Supplementary file 2 — Additional file 2: Figure S2. Effect of various concentrations of salt (NaCl) on cotton plant (stress time duration was 3 h). [file 12864_2020_7260_MOESM2_ESM.docx]

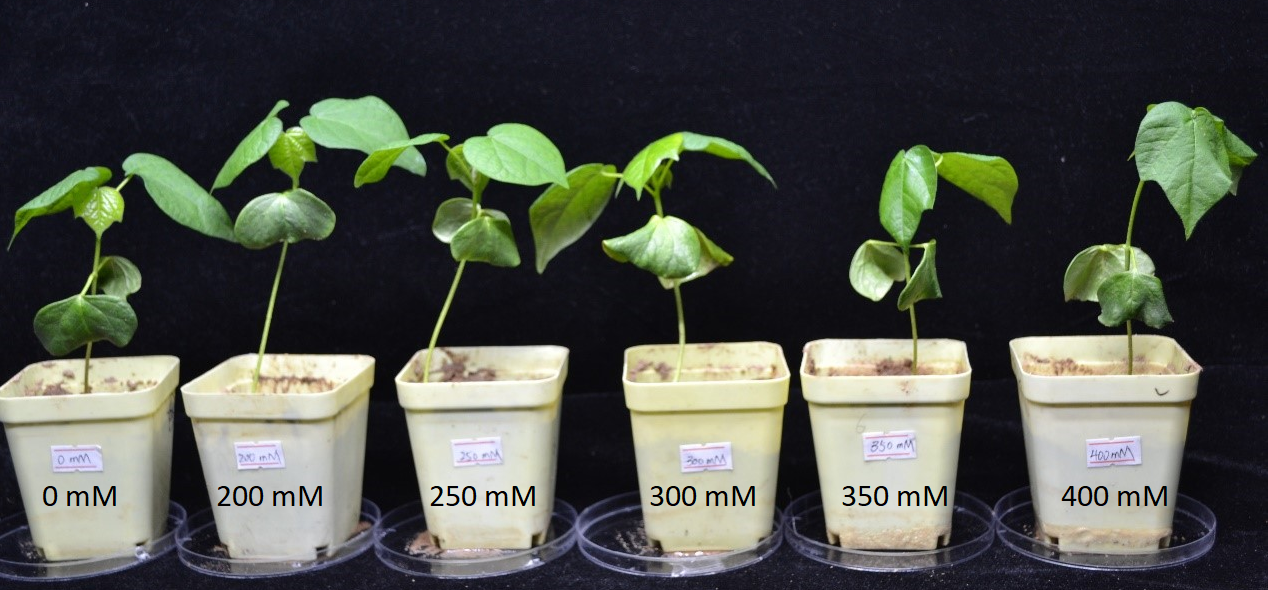


**Figure S2**. Effect of various concentrations of salt (NaCl) on cotton plant (stress time duration was 3 h).
